# Factors contributing to antibiotic misuse among parents of school-going children in Dhaka City, Bangladesh

**DOI:** 10.1038/s41598-024-52313-y

**Published:** 2024-01-28

**Authors:** Md Wahidul Islam, Muhibullah Shahjahan, Abul Kalam Azad, Md Jubayer Hossain

**Affiliations:** 1 Population Health Studies Division, Center for Health Innovation, Research, Action, and Learning-Bangladesh (CHIRAL Bangladesh), 9-10 Chittaranjan Ave, Dhaka, 1100 Bangladesh; 2 Department of Microbiology, Jagannath University, 9-10 Chittaranjan Ave, Dhaka, 1100 Bangladesh

**Keywords:** Antimicrobials, Applied microbiology, Bacteriology, Microbial communities, Environmental microbiology, Infectious-disease diagnostics, Policy and public health in microbiology

## Abstract

Antimicrobial resistance (AMR) is a pressing global health concern, especially in resource-constrained countries, such as Bangladesh. This study aimed to identify the factors contributing to antibiotic misuse by assessing knowledge, attitude, and practice (KAP). A cross-sectional study was conducted from August 20 to August 30, 2022, among 704 parents of school-going children in Dhaka South City. Descriptive statistics were used to analyze the KAP, and multivariate models, including linear and ordinal logistic regression, were used to explore the associations between these factors. The findings revealed that approximately 22% of the participants were male and 78% were female. Most parents (58%) had completed higher secondary education. Approximately 45% of the respondents demonstrated moderate knowledge, 53% had uncertain attitudes, and 64% exhibited antibiotic misuse. Factors such as parental age, education level, employment status, income, child's age, and family type significantly influenced KAP. These findings emphasize the importance of targeted education and awareness initiatives to enhance knowledge and responsible antibiotic use among parents, contributing to global efforts against antibiotic resistance. The government should enforce laws and regulations regarding the misuse of antibiotics.

## Introduction

Antimicrobial resistance (AMR) is recognized as a pressing public health issue with long-term and unpredictable health, social, and economic impacts worldwide^1–5^. AMR has a wide range of effects, including prolonged hospital stay, financial burden, morbidity, and mortality^6–8^. The World Health Organization (WHO) reported in 2019 that approximately 700,000 annual deaths globally were attributed to AMR. It is projected that by 2030, AMR will contribute to an estimated 10 million deaths worldwide^4,9^. Interestingly, a proportional relationship was observed between antibiotic use and the development of AMR. Antibiotic consumption and AMR have continuously increased. Recent research has shown that human antibiotic consumption amounted to approximately 34.8 billion doses annually, with a 65% increase in global usage between 2000 and 2015. This rise in antibiotic consumption contributes to the global spread of antibiotic-resistant bacterial infections^10–13^. The WHO is concerned that AMR could lead to a post-antimicrobial era in which common infections could become deadly.

The burden of antimicrobial resistance (AMR) is significantly higher in developing nations due to excessive antibiotic prescriptions, insufficient patient education, incomplete medication courses, antibiotic overuse in animal and fish farming, subpar infection control in healthcare facilities, inadequate hygiene and sanitation practices, unregulated antibiotic sales, and a dearth of new antibiotic development^14–17^. The emergence and spread of AMR have been recognized by the WHO as a hotspot among developing countries in Southeast Asia (SEA). The extensive use of antibiotics in Southeast Asia across various sectors, including humans, animals, aquaculture, and agriculture, is primarily attributed to their widespread usage^18–22^. In Bangladesh, a country in Southeast Asia, the use of antibiotics is increasing and there is a lack of effective regulations governing their use^12,23–29^. Consequently, the prevalence of AMR bacteria-associated infections is increasing^30^. According to a 2018 report, superbugs and bacteria resistant to multiple drugs were identified in approximately 14% of all infection cases, and approximately 70% of fatalities in intensive care units resulted from infections resistant to multiple drugs^28^. Notably, this issue affects individuals in all age groups. However, the incidence of AMR infections is higher among children in developing nations such as Bangladesh^28,31^.

Emerging from Southeast Asia, Bangladesh presents a regional and global challenge owing to its high prevalence of Antimicrobial Resistance (AMR)^32^. This increase in antimicrobial resistance (AMR) is directly linked to widespread self-medication practices within the Bangladeshi population^33^. Unregulated access to antibiotics, evidenced by an alarming 18% presence in Dhaka households without prescriptions, fuels rampant self-medication for minor ailments, amplifies the rise of Antimicrobial Resistance (AMR), and poses dire individual and societal threats^34^. Between 2016 and 2018, antibiotic consumption witnessed a precipitous surge from 16.6 to 21.8%, further exacerbating the crisis^35^. A comprehensive review revealed alarmingly high resistance rates against a multitude of tested microorganisms, effectively rendering many first-line antibiotics therapeutically inefficacious^32^. Despite commendable national drug policies and reasonably good public healthcare access in Bangladesh, a significant proportion of antimicrobials are consumed without doctor prescriptions^24,27,36^. This ubiquitous self-medication, often orchestrated through community pharmacies, bypasses established national guidelines and perpetuates antimicrobial overuse in low- and middle-income countries (LMICs) such as Bangladesh^22,37^.

Globally, children are vulnerable to infections and use substantial quantities of antibiotics^14,38–40^. Several factors, including excessive prescription of antibiotics by physicians, parents' limited understanding of how to use antibiotics, and the ease of obtaining antibiotics for self-medication have been proposed as contributing factors to inappropriate use of antibiotics in children^41–44^. Antimicrobial resistance is strongly linked to individuals' knowledge, attitudes, and behaviors concerning the use of antibiotics^45^. A strong correlation exists between insufficient knowledge of antibiotic usage and antimicrobial resistance (AMR) as well as higher antibiotic consumption within communities^44–50^. Hence, it is crucial to comprehend the knowledge, attitudes, and behaviors of parents concerning antibiotic usage as well as the prescribing habits of doctors. This will enable the development of effective interventions aimed at enhancing the use of antibiotics.

This study aimed to investigate parents’ knowledge, attitudes, and practices (KAP) regarding antibiotic misuse and resistance among school-going children in Dhaka, South City. This study hypothesized that there is a significant knowledge gap among parents in Dhaka regarding appropriate antibiotic use, contributing to instances of misuse. Furthermore, our hypothesis suggests that parental perspectives shape antibiotic administration practices, emphasizing the importance of understanding these subjective factors. Finally, the investigation aimed to identify factors associated with inappropriate antibiotic use, including sociocultural, economic, and healthcare system-related factors. This study sought to provide insights into parental behaviors and beliefs, which could inform targeted interventions and public health initiatives to mitigate antibiotic misuse and resistance in the study population.

## Methods

### Study design and study participants

A community-based cross-sectional study was conducted to determine the level of Knowledge, Attitude and Practice (KAP) and factors associated with the misuse of antibiotics in children among parents at 10 schools in Dhaka City, Bangladesh. These schools were located at the Dhaka South City Corporation. The study areas were categorized as private and government schools in Dhaka Old City. The selection of schools within each zone was based on the type of school, whether private or government. Participants were randomly selected from within each school. This study was conducted between August and September 2022. The study area was located at the Dhaka South City Corporation.

### Sample size

The required sample size was calculated using the following equation^51,52^:

$$n= \frac{{z}^{2 }pq}{{d}^{2}}$$

n = number of samples, z = 1.96 (95% confidence level), p = prevalence estimate (50% or 0.5); as no study found in Bangladesh, q = (1 − p), d = precision limit or proportion of sampling error (5% or 0.05), So, n = 1.96^2^ × 0.5 × (1 − 0.5)/0.05^2^ ~ 384.16.

The rationale for employing this formula is rooted in its capacity to balance statistical robustness with practical feasibility. The conservative assumption of 50% prevalence ensures the adequacy of the calculated sample size across a spectrum of prevalence scenarios. The selection of a 95% confidence level, with an associated Z-score of 1.96, adheres to customary epidemiological standards, instilling a high degree of confidence in the precision of our estimates. Given the focus of our study on parental knowledge, attitudes, and practices related to antibiotic misuse and resistance in a specific urban context (Dhaka, South City), the chosen sample size was designed to accommodate potential variations within the target population, while maintaining statistical power. This approach allows for a comprehensive exploration of the prevalence of antibiotic misuse and the associated factors among parents. Notably, the calculated minimum sample size of 384 surpasses this requirement, reinforcing the reliability of our findings. Grounded in established statistical principles and tailored to the distinctive features of our study, this methodological approach to sample-size determination contributes to the overall academic rigor and credibility of our research.

### Survey instrument

A 42-item structured questionnaire was developed based on an extensive literature review of studies highlighting the influence of KAP on the irrational use of antibiotics in children^36,39,45–49^. Some of the questions were adopted, while others were customized to suit parents (assuming that most of the parents were non-medical professionals and the level of education was not equal). The questionnaire was prepared in English and translated into Bengali to facilitate convenient and accurate data collection. The research team verified the accuracy and meaning of the translated content. The questionnaire was first validated and tested to answer the study objectives by randomly selecting ten respondents in a pilot study conducted in Azimpur between July 1 and July 5, 2022.

### Types of data collected

The data were collected using structured questionnaires. The questionnaire consisted of five sections. Section I: Collected data on sociodemographic information of the study participants (parents), including age, gender, family type, number of children, educational qualification, marital status, and residence, as well as sociodemographic information of the child, such as age and sex. Section II consisted of questions on knowledge of antibiotic use. Section III comprises questions on the influence of attitudes toward the use of antibiotics. Section IV consisted of questions assessing parental practices regarding the appropriate use of antibiotics. Section V consisted of questions on parents’ sources of information about antibiotics.

### Data collection

A field data collector team completed the data collection under the guidance of lead researchers in this study. These data collectors received training in investigation skills and research ethics from public health specialists and microbiologists specializing in antibiotic use and misuse at the Center for Health Innovation, Research, Action, and Learning, Bangladesh (CHIRAL Bangladesh). The data collection team was responsible for inviting participants and guiding the completion of the questionnaire, following a standard data collection procedure. Face-to-face interviews were conducted to collect information, and the interviews were completed within 20–30 min.

### The reliability of the questionnaire

To validate and ensure the reliability of our data collection tool, we conducted a comprehensive evaluation of the questionnaire's validity and internal consistency. We maintained strict adherence to the content validity through an iterative process that involved collaboration with medical experts. The internal consistency of the questionnaire was assessed using Cronbach's alpha values calculated using the psych R package^56^. The alpha value for each section (Knowledge, Attitude, and Practices) exceeded 0.7, confirming the questionnaire's robustness in measuring the intended constructs. Ethical considerations were carefully addressed, with informed consent obtained from participants. Ethical approval for this study was obtained from the Bangladesh Center for Health, Innovation, Research, Action, and Learning Ethical Review Committee (reference number: CHIBAN20AUG2022-0004). The chief of the schools requested permission to conduct this study. Written informed consent was obtained from parents after explaining the purpose of the study before enrollment. This study was conducted in accordance with the laws and regulations of Bangladesh and followed the Declaration of Helsinki.

### Data analysis

Data was collected using an Open Data Kit (ODK software, KoboCollect). Questionnaires with responses recorded on more than 90% of the questions were considered well completed and were included in the statistical analysis using R Programming (version 4.2.1). Reproducible descriptive tables were created using the gtsummary-R package for reproducible research^57^. Categorical variables, such as sex, education level, and marital status, are presented as frequencies and percentages. Means and standard deviations were used to summarize the numeric variables. A particular question was categorized as correct/uncertain/incorrect for knowledge during data presentation, agree/disagree/uncertain for attitude, or yes/no for practice.

Overall knowledge, attitudes, and practices have been estimated previously^45,49^. For every question, the correct response was scored 1, whereas incorrect answers and uncertainty were scored 0. Twelve questions were used to assess parents’ knowledge of antibiotics, ten to determine parents’ attitudes, and six to evaluate parents’ practices. The proportion of respondents who answered each question correctly was then calculated. To describe the general distribution of awareness, a score for antibiotic knowledge (0–12), attitude (0–10), and practice (0–6) was created for each parent, based on the number of correct answers. The median (50%) score was used as the cut-off to dichotomize this continuous variable as the dependent variable in simple and multiple logistic regressions. Parents scoring higher than the median were assessed as having better knowledge, a positive attitude, and a good practice of antibiotic use. Depending on the score, knowledge was categorized as poor, moderate, or good, whereas attitude was categorized as negative, uncertain, or positive, respectively. Poor/negative was given a score of 0–49%, moderate/uncertain was given a score of 50–79%, and good/positive was 80–100%. In this study, questions on practice were used to measure the appropriate or inappropriate use of antibiotics. The percentage score for practice was inappropriate (misuse) if the score was 0–79% and appropriate (good use) if the score was 80–100%.

The score is calculated as the percentage of correct responses to the total number of questions in each category (K/A/P). An ordinal logistic regression model was used to determine factors associated with positive attitudes and knowledge. The covariates and potential confounders included in the model were carefully chosen to ensure a comprehensive analysis of the factors influencing knowledge, attitude, and practice regarding antibiotic use. Beyond demographic variables, such as parents' age, sex, and education level, we incorporated socioeconomic factors, such as employment status and household income. Recognizing the significance of family dynamics, we also included family type and grandparents’ involvement in treatment decisions. The model further incorporates a set of variables related to participants' knowledge, attitudes, and practices towards antibiotics, as well as their self-reported practices regarding antibiotic use. These additional variables aim to account for potential confounding effects and provide a better understanding of the factors that influence knowledge outcomes. The detailed model specification enhances the transparency and rigor of our statistical analysis, contributing to the overall robustness of our study.

Binary logistic regression analysis was conducted to determine the factors associated with good practices. The measure of association was the odds ratio (OR) for univariate and multivariate analyses. A p-value of less than 0.05 at a confidence interval of 95% (95% CI) indicated statistical significance.

## Results

### Demographic characteristics of the participants

A total of 704 respondents participated in the study, of which 153 (22%) were male and 551 (78%) were female. Parents’ ages were classified into four categories: < 25, 25–35, 36–45, and greater than 45 years (approximately 1.8%, 54%, 38%, and 6.7%, respectively). The educational status of parents was diverse; most parents had completed higher secondary education (54%), followed by postgraduate (25%), undergraduate (16%), and primary education (5%). Among the respondents, 71.5% were unemployed, and 28.5% were employees. Most participants belonged to nuclear families (53%), followed by single-parent families (26%) and extended families (21%), while the majority (58%) belonged to middle-income families. Of the total respondents, 54% had female children, while the rest of the participants in the study had male children; 60% of parents had only two children, 25% had only one, and 15% had more than two children. Approximately 89% of parents reported that the mother was the leading caregiver of the child at home. In the case of disease treatment, 65% said that their child's grandparents were not involved in decision making, whereas 35% reported that they were involved in decision making when their child was ill (Table 1).

**Table 1:** Demographic characteristics of study participants (N = 704).

Characteristic	N = 704
Parent’s age (years)	
< 25	13 (1.8%)
> 45	47 (6.7%)
25–35	377 (54%)
36–45	267 (38%)
Parent’s sex	
Female	551 (78%)
Male	153 (22%)
Parent’s education level	
Postgraduate	175 (25%)
Primary	35 (5.0%)
Secondary	381 (54%)
Undergraduate	113 (16%)
Employment status	
Employed	95 (13%)
Not employed	503 (71%)
Self employed	106 (15%)
Family type	
Extended family	147 (21%)
Nuclear family	372 (53%)
Single parent family	185 (26%)
Your average household income per month (USD)	
High (greater than 550 USD)	139 (20%)
Low (less than 350 USD)	160 (23%)
Middle (less than 550 USD)	405 (58%)
Child’s sex	
Female	379 (54%)
Male	325 (46%)
Child’s age (years)	
< 5	37 (5.3%)
> 10	313 (45%)
5–9	353 (50%)
Number of children	
> = 3	104 (15%)
1	176 (25%)
2	424 (60%)
Who is the leading child caregiver at home?	
Father	54 (7.7%)
Grandmother	16 (2.3%)
Mother	629 (89%)
Others	5 (0.7%)
Are grandparents at home involved in treatment decisions when your child is ill?	
Always	34 (5%)
Never	459 (65%)
Often	211 (30%)

### Distribution of knowledge regarding antibiotic resistance

**Figure 1:**
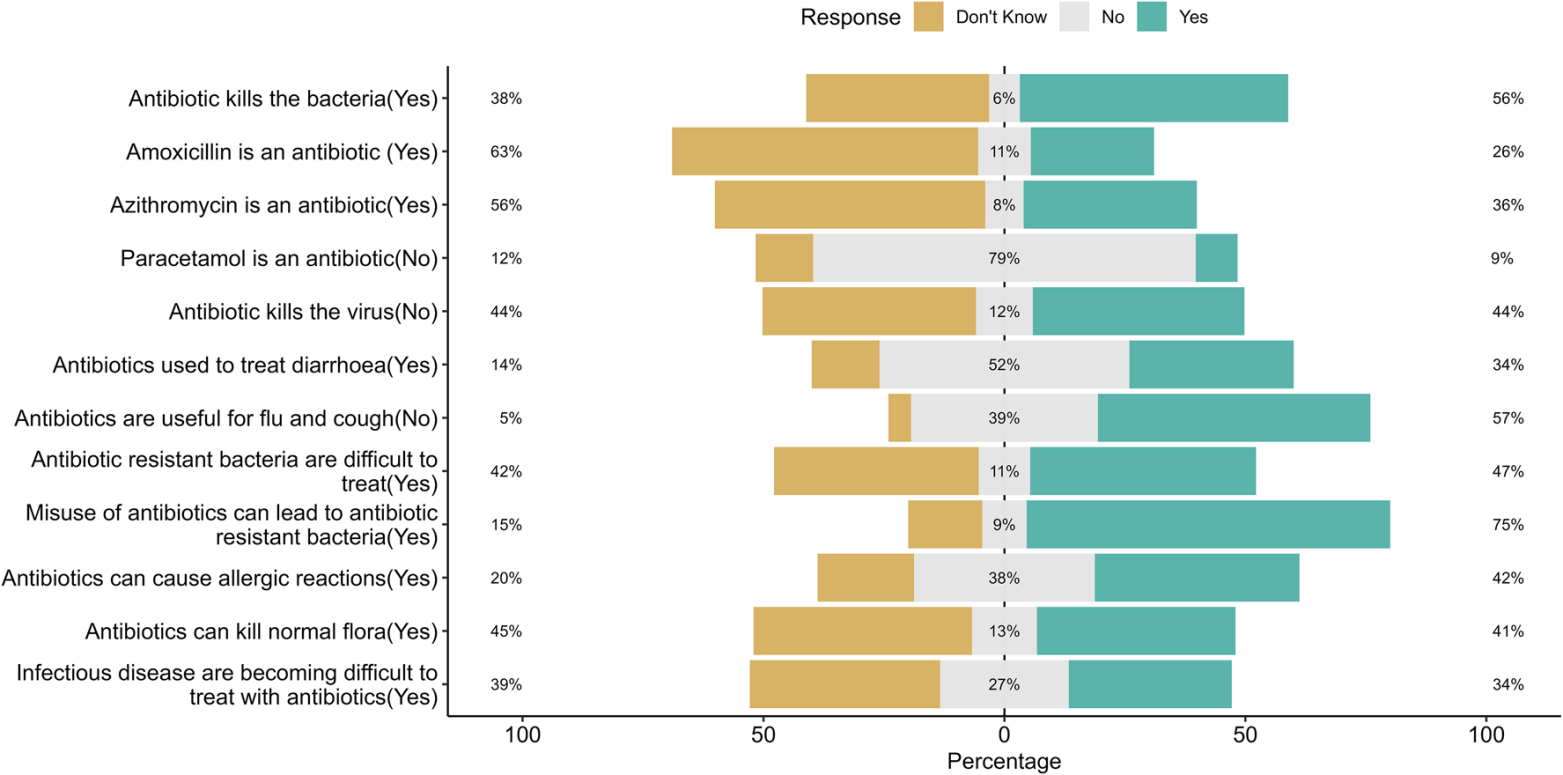
Distribution of knowledge of antibiotic resistance among parents of school-going children (N = 704).

Figure 1 provides the distribution of knowledge regarding antibiotics, a significant proportion of the participants showed a lack of knowledge in recognizing the basic antibiotics of which 63% and 56% did not know that amoxicillin and azithromycin were antibiotics, respectively, while 79% knew that paracetamol was not an antibiotic. Of most participants, 75% knew that the misuse of antibiotics could lead to AR, and approximately 47% believed that antibiotic-resistant bacteria are difficult to treat.

### Attitude towards misuse of antibiotics

**Figure 2:**
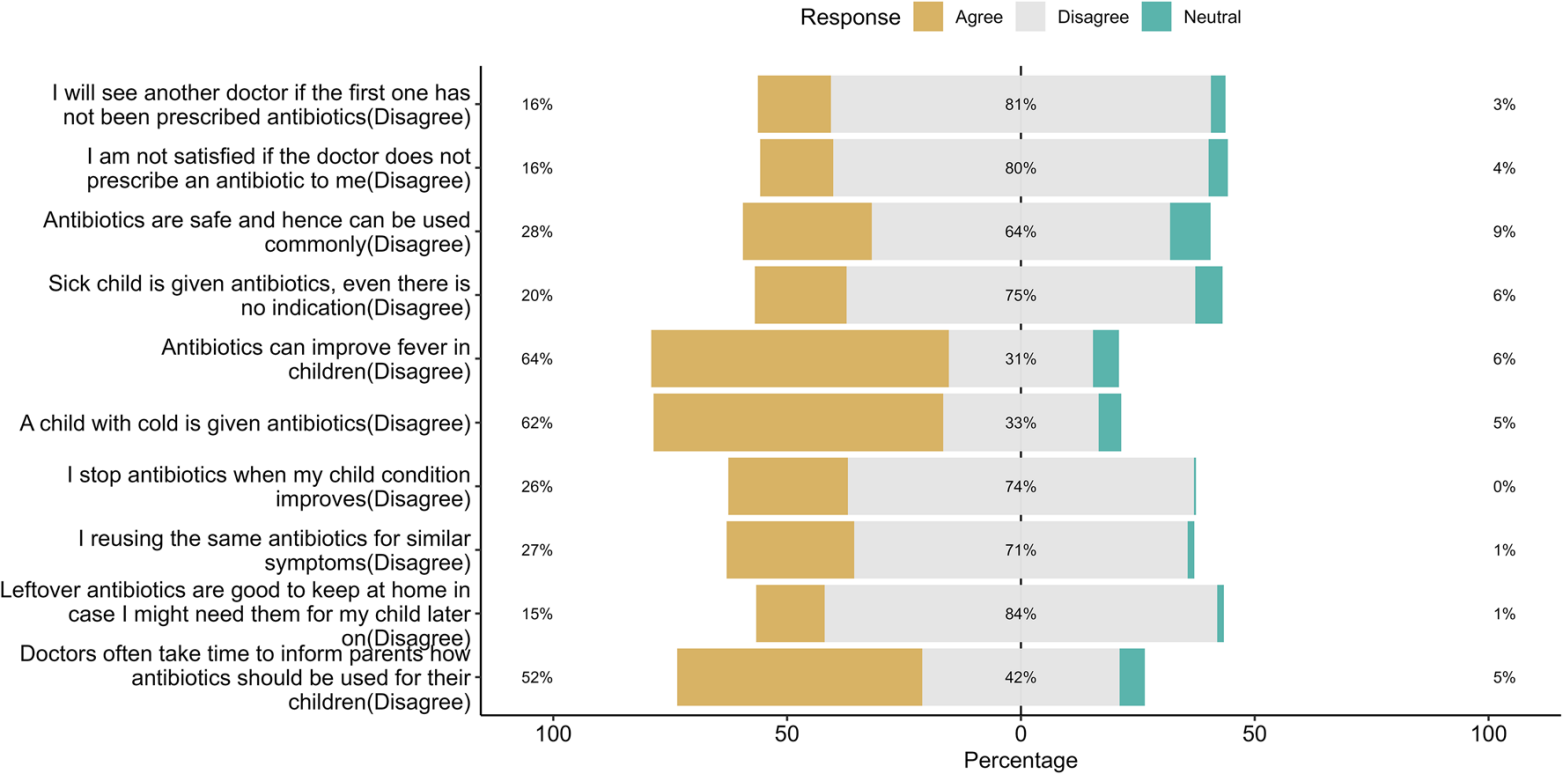
Attitude towards antibiotic resistance and the misuse of antibiotics among parents of school-going children (N = 704).

Figure 2 shows the distribution of parents’ attitudes toward the misuse of antibiotics. More than 80% had a positive attitude toward non-antibiotic prescriptions and were satisfied with the doctor’s prescription. In contrast, 75% disagreed with the provision of antibiotics to their children without indication. The majority of parents (approximately 63%) believed that antibiotics could be used for fever and cold improvement. Additionally, some parents 26% were ready to stop administering antibiotics to their children when there were improvements and 27% reused the same antibiotics for similar symptoms.

### Practices regarding the use of antibiotics

**Figure 3:**
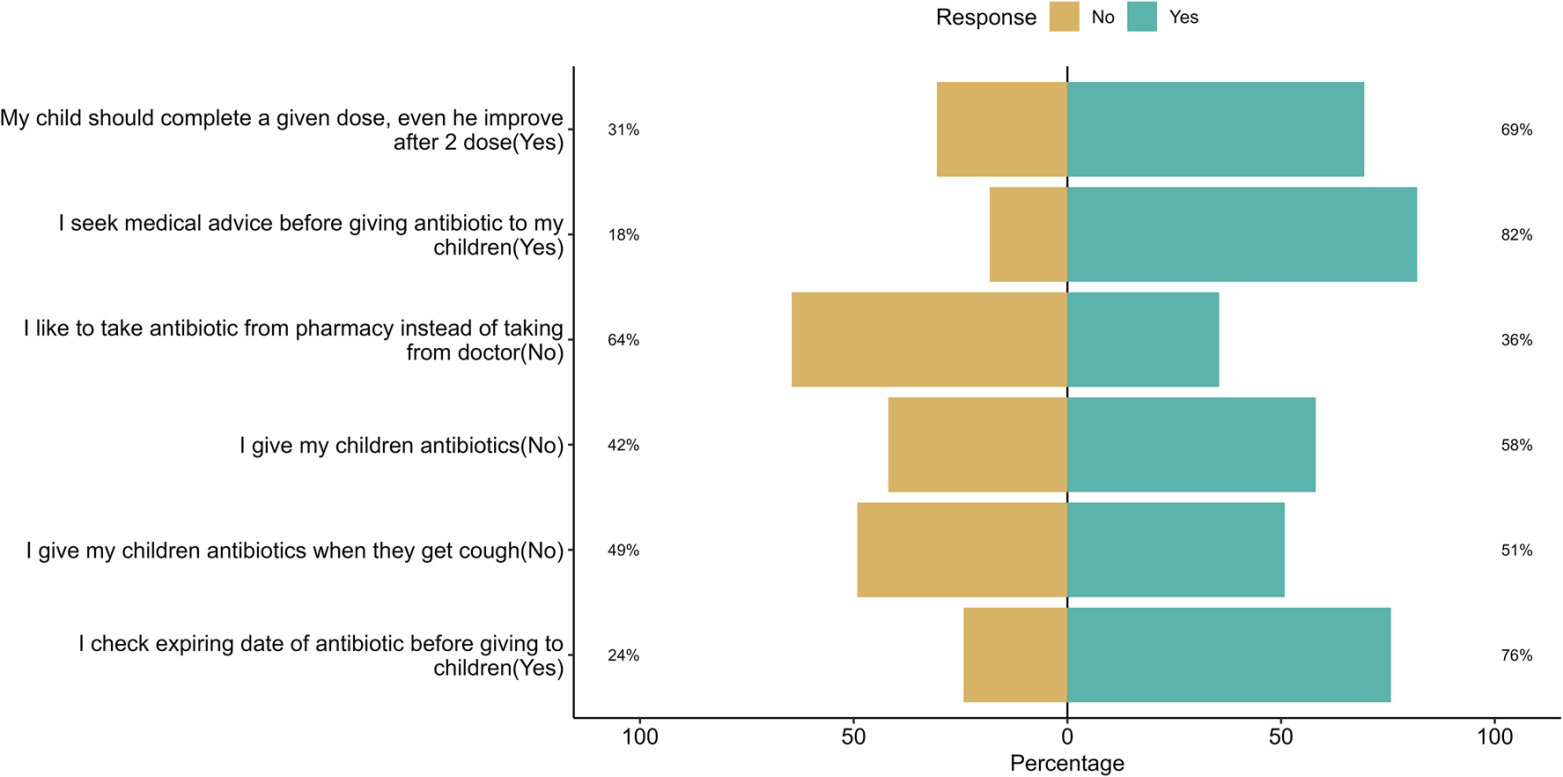
Practices among parents of school-going children regarding antibiotic resistance (N = 704).

The majority (58%) of respondents gave antibiotics to their children without consulting a doctor, and 36% liked taking antibiotics from pharmacies rather than from doctors. Approximately 51% of the parents gave antibiotics to their children when they had a cough. Regarding the expiry date, 24% reported that they did not check the expiration date of antibiotics before giving them to their children (Fig. 3).

### Major sources of information regarding antibiotics

Most parents obtained their information from prescribers (86%), followed by dispensers (36%) and the Internet (30%). Few parents obtained information about antibiotics from social media (23%), pharmaceutical companies (11%), or other sources (23%) including colleagues, nurses, and university courses. (Table 2).

**Table 2:** Major sources of information about antibiotic parents (N = 704).

Characteristic	N = 704
Information provided by pharmaceutical companies' leaflet	78 (11%)
Information from prescribers	607 (86%)
Information from dispensers	252 (36%)
Information from nurses	22 (3.1%)
Information given by a colleague	34 (4.8%)
Information from University courses	16 (2.3%)
Internet	213 (30%)
Social media	165 (23%)
Others	89 (13%)

### Level of knowledge, attitudes, and practices regarding antibiotic resistance

Overall, the level of knowledge, attitudes, and practices regarding rational use of antibiotics in children. Of the 704 parents assessed on the KAP, 17% (n = 122) had good and 45% (n = 314) had moderate knowledge, 30% (n = 209) had a positive attitude, and 36% (n = 250) had good practices on rational antibiotic use in children (Table 3).

**Table 3:** Level of knowledge, attitudes, and practices towards antibiotic resistance among parents with school-going children (N = 704).

Characteristic	N = 704
Knowledge level	
Good	122 (17%)
Moderate	314 (45%)
Poor	268 (38%)
Attitude level	
Positive	209 (30%)
Negative	124 (18%)
Uncertain	371 (53%)
Practice level	
Good	250 (36%)
Misuse	454 (64%)

### Factors associated with level of knowledge regarding antibiotic resistance

Ordinal logistic regression analysis was conducted to predict the factors associated with the level of knowledge regarding antibiotic resistance among parents (Table 4). The findings revealed that parents aged 25–35 years (OR = 0.20, 95% CI 0.04–0.70, *p* = 0.020) and 36–45 years (OR = 0.16, 95% CI 0.03–0.56, *p* = 0.008) had significantly lower odds of having knowledge about antibiotic resistance compared to those over 45 years of age. On the other hand, parents with primary (OR = 6.84, 95% CI 3.15–15.7, *p* < 0.001) and secondary education (OR = 2.49, 95% CI 1.71–3.62, *p* < 0.001) exhibited significantly higher odds of knowledge than postgraduates. Not being employed (OR = 2.28, 95% CI 1.26–4.13, *p* = 0.007) was associated with increased odds of knowledge compared to employment. Moreover, having a low household income (less than 350 USD) (OR = 2.28, 95% CI 1.43–3.67, *p* < 0.001) was associated with higher odds of knowledge than high income levels. Children aged 5–9 years (OR = 2.56, 95% CI 1.31–5.05,* p* = 0.006) and over 10 years (OR = 2.48, 95% CI 1.25–4.98, *p* = 0.010) had significantly higher odds of knowledgeable parents than those under 5 years old. In contrast, factors such as parents’ sex, undergraduate education level, family type, children’s sex, and the number of children did not show significant associations with knowledge regarding antibiotic resistance.

**Table 4:** Factors associated with the level of knowledge among parents of school-going children (N = 704).

Characteristic	OR	95% CI	*p*-value
Parent’s age (years)			
< 25	–	–	
> 45	0.18	0.03, 0.75	0.026*
25–35	0.20	0.04, 0.70	0.020*
36–45	0.16	0.03, 0.56	0.008*
Parent’s sex			
Female	–	–	
Male	1.28	0.73, 2.28	0.4
Parent’s education level			
Postgraduate	–	–	
Primary	6.84	3.15, 15.7	< 0.001*
Secondary	2.49	1.71, 3.62	< 0.001*
Undergraduate	1.43	0.89, 2.29	0.14
Employment status			
Employed	–	–	
Not employed	2.28	1.26, 4.13	0.007*
Self employed	0.91	0.53, 1.57	0.7
Family type			
Extended family	–	–	
Nuclear family	1.04	0.71, 1.52	0.8
Single parent family	1.07	0.69, 1.66	0.8
Your average household income per month (USD)			
High (greater than 550 USD)	–	–	
Low (less than 350 USD)	2.28	1.43, 3.67	< 0.001*
Middle (less than 550 USD)	1.45	0.99, 2.13	0.056
Child’s sex			
Female	–	–	
Male	0.92	0.69, 1.24	0.6
Child’s age (years)			
< 5	–	–	
> 10	2.48	1.25, 4.98	0.010*
5–9	2.56	1.31, 5.05	0.006*
Number of children			
> = 3	–	–	
1	1.04	0.63, 1.71	0.9
2	0.95	0.62, 1.45	0.8

*OR* odds ratio, *CI* confidence interval. *p-value ˂ 0.05 was considered statistically significant.

Significant values are in bold.

### Factors associated with level of attitudes towards antibiotic resistance

Ordinal logistic regression was conducted to assess the factors associated with participants’ level of knowledge (Table 5). None of the parents’ age groups (i.e., < 25, 25–35, 36–45, and > 45 years) showed statistically significant associations with their attitudes towards antibiotic resistance. Similarly, parents’ educational levels, including primary and undergraduate education, did not significantly impact their attitudes. Employment status also did not have a significant association, although not being employed, showed a trend towards increased positive attitudes (OR = 1.62, 95% CI 0.91–2.87,* p* = 0.10). Male parents were significantly more likely to exhibit positive attitudes towards antibiotic resistance (OR = 2.40, 95% CI 1.37–4.26,* p* = 0.002) than female parents. Family type, household income, sex, age, and number of children did not show statistically significant associations with parents' attitudes towards antibiotic resistance.

**Table 5:** Factors associated with the level of attitudes towards antibiotic resistance among parents of school-going children (N = 704).

Characteristic	OR	95% CI	p-value
Parent’s age (years)			
< 25	–	–	
> 45	0.54	0.15, 1.92	0.3
25–35	0.86	0.27, 2.53	0.8
36–45	0.64	0.20, 1.96	0.4
Parent’s sex			
Female	–	–	
Male	2.40	1.37, 4.26	0.002*
Parent’s education level			
Postgraduate	–	–	
Primary	0.87	0.40, 1.91	0.7
Secondary	0.61	0.42, 0.89	0.010*
Undergraduate	0.80	0.50, 1.28	0.3
Employment status			
Employed	–	–	
Not employed	1.62	0.91, 2.87	0.10
Self employed	0.80	0.46, 1.38	0.4
Family type			
Extended family	–	–	
Nuclear family	1.03	0.71, 1.49	0.9
Single parent family	0.81	0.53, 1.23	0.3
Your average household income per month (USD)			
High (greater than 550 USD)	–	–	
Low (less than 350 USD)	0.75	0.48, 1.19	0.2
Middle (less than 550 USD)	1.03	0.70, 1.49	0.9
Child’s sex			
Female	–	–	
Male	1.15	0.86, 1.55	0.3
Child’s age (years)			
< 5	–	–	
> 10	0.94	0.45, 1.88	0.9
5–9	0.86	0.42, 1.68	0.7
Number of children			
> = 3	–	–	
1	1.02	0.62, 1.67	> 0.9
2	1.11	0.73, 1.68	0.6

*OR* odds ratio, *CI* confidence interval. *p-value ˂ 0.05 was considered statistically significant.

Significant values are in bold.

### Factors associated with the level of practice towards the misuse of antibiotics

A binary logistic regression model was used to predict the level of practice using the demographic variables. Table 6 provides a comprehensive overview of the outcomes derived from binary logistic regression, offering valuable insights into the factors that play a role in shaping specific practices, particularly in the context of antibiotic use among parents. Notably, households with lower monthly incomes (less than 350 USD) and those classified as middle-income (less than 550 USD) showed significantly higher odds of practicing antibiotic use than high-income households (OR = 2.76, 95% CI 1.61–4.80,* p* < 0.001; OR = 3.01, 95% CI 1.95–4.68,* p* < 0.001, respectively). Additionally, positive attitudes were strongly associated with better antibiotic use practices, as evidenced by notably lower odds of practicing antibiotic use among individuals with a positive attitude (OR = 0.11, 95% CI 0.05–0.21, *p* < 0.001).

**Table 6:** Factors associated with the level of practices regarding antibiotic resistance among parents of school-going children (N = 704).

Characteristic	OR	95% CI	*p-*value
Parent’s age (years)			
< 25	–	–	
> 45	0.11	0.01, 0.57	0.016*
25–35	0.24	0.03, 1.01	0.081
36–45	0.25	0.04, 1.11	0.10
Parent’s sex			
Female	–	–	
Male	0.96	0.47, 1.93	> 0.9
Parent’s education level			
Postgraduate	–	–	
Primary	1.52	0.60, 4.19	0.4
Secondary	0.81	0.52, 1.26	0.4
Undergraduate	0.65	0.37, 1.12	0.12
Employment status			
Employed	–	–	
Not employed	1.00	0.49, 2.03	> 0.9
Self employed	1.71	0.88, 3.37	0.12
Family type			
Extended family	–	–	
Nuclear family	0.89	0.57, 1.38	0.6
Single parent family	1.03	0.62, 1.70	> 0.9
Your average household income per month (USD)			
High (greater than 550 USD)	–	–	
Low (less than 350 USD)	2.76	1.61, 4.80	< 0.001*
Middle (less than 550 USD)	3.01	1.95, 4.68	< 0.001*
Child’s sex			
Female	–	–	
Male	1.34	0.95, 1.89	0.10
Child’s age (years)			
< 5	–	–	
> 10	0.62	0.26, 1.41	0.3
5–9	0.76	0.33, 1.69	0.5
Number of children			
> = 3	–	–	
1	0.72	0.39, 1.30	0.3
2	0.93	0.56, 1.53	0.8
Knowledge level			
Good	–	–	
Moderate	0.85	0.52, 1.37	0.5
Poor	1.02	0.60, 1.73	> 0.9
Attitude level			
Negative	–	–	
Positive	0.11	0.05, 0.21	< 0.001*
Uncertain	0.23	0.12, 0.42	< 0.001*

*OR* odds ratio, *CI* confidence interval. *p value ˂ 0.05 was considered statistically significant.

Significant values are in bold.

## Discussion

The prominent outcomes of this study were poor (38%) to moderate (45%) knowledge, negative attitudes (71%, including 53% uncertainty), and misuse (64%) of antibiotics among the parent participants, which is not a satisfactory and serious public health concern in developing countries, such as Bangladesh.

To our knowledge, this is the first broad-range KAP-based study in Bangladesh on the misuse of antibiotics by parents of school-going children. Though, there are several studies conducted regarding antibiotic uses and misuses in Bangladesh^33,58–61^.

The results of the knowledge section indicated that parents were not knowledgeable about antibiotic usage. Nearly half (45%) had moderate knowledge and 38% had poor knowledge, indicating poor knowledge about antibiotic use. Most (45%) had misunderstandings regarding antibiotic identification and use, and there was an erroneous misconception that antibiotics could treat viral infections. Previous studies have reported similar results^31,41,62–66^. Another novel finding is that approximately 75% and 47% of parents are aware that the misuse of antibiotics can lead to AR, and antibiotic-resistant bacteria are difficult to treat, respectively, which is similar to earlier findings in studies conducted in comparatively more developed nations such as Saudi Arabia (96%), Cyprus (90%), Greece (88%), Sweden (81%), Israel (78%), and India^53,67–72^.

This study identified several factors related to strong parenting knowledge of antibiotic use in children, including parents’ age, educational level, employment status, household income, and children’s age (*p* < 0.05). Older parents (> 25 years) had significantly better knowledge of antibiotic use in their children than younger parents did because of their parenting age and previous experiences of common illness. Similar findings have been previously reported^19,70,73,74^. Formal education (primary and secondary) was strongly associated with good knowledge about antibiotic use in children rather than undergraduate and postgraduate education, which may be because the leading caregivers of children were mothers (89%), and they were the least likely to know about antibiotic-resistant bacteria. Similar conclusions were reached in several countries, including India, China, and Denmark^42,55,67,75^.

Parental attitudes toward antibiotic use have a major influence on the irrational use of antibiotics, resulting in the development of AR in children^76^. Approximately 18% had a negative attitude; furthermore, 53% of parents had uncertain opinions regarding antibiotic use. More than half of the participants (64% and 62%) had a positive attitude toward using antibiotics for fever and the common cold, respectively, for their children without a doctor’s consultation, which is a major concern in developing countries such as Bangladesh. Similar results have been reported in studies Tanzania, Jordan, and China have reported similar results^6,27,28^. Surprisingly, approximately 30% of parents had a positive attitude regarding antibiotic use, while more than 80% showed a positive attitude by disagreeing with the idea that leftover antibiotics should be kept at home for later use. Additionally, more than 80% of parents were satisfied with the doctor's prescription and had a favorable opinion regarding antibiotic-free medicines. In summary, these findings are consistent with those of previous studies^31,53,67,77^.

In this study, parents’ sex and education level were significantly associated with positive attitudes toward children's antibiotic usage. Male parents and secondary-educated parents showed more positive attitudes than other groups, which is consistent with previous research conducted in the UAE and Tanzania on parents’ education levels^22,27^. Surprisingly, parental gender was significantly associated with positive attitudes in the present study; these results contradict the claims of previous studies conducted in the UAE, Tanzania, and Qatar^39,61,67^. Several factors could explain this observation. First, previous studies were conducted in developed areas, and second, due to differences in the sample size. First, previous studies were conducted in developed areas, and second, due to the differences in sample size.

Parental practice has a major influence on the irrational use of antibiotics, resulting in the development of AR in children. The present study confirmed the findings regarding misuse among 64% of parents; more than half (58%) of parents were willing to give their children antibiotics without a doctor's prescription, and 36% favored buying their antibiotics from pharmacies rather than from doctors. Therefore, it is easier for parents to purchase antibiotics from pharmacies in countries without prescriptions, such as Bangladesh, leading to an increase in irrational antibiotic use in children. A similar pattern of results was obtained in previous studies^31,45,78^. Furthermore, approximately 51% of parents administered antibiotics as treatment to their children for common colds, fevers, and coughs. The similar findings have been observed in previous KAP investigations conducted in Malaysia, Tanzania, and Palestine^45,54,71^.

Another important finding was that 69% of parents completed the antibiotic dose of their children during medication, 82% sought medical advice from doctors before administering antibiotics, and 76% checked the expiration date of antibiotics. This finding provides a clear understanding that 36% of parents had good antibiotic practice. The observed percentage was slightly higher than that reported in a previous study conducted in Tanzania (18%) with a larger sample size in India (33%)^45,67^. The present study found a significant correlation between good practices and several factors, including parents’ age, lower household income, and positive attitudes toward uncertainty. Positive and uncertain attitudes of parents contribute to good antibiotic practices and help reduce antibiotic resistance^5,27,33,34^.

Weak surveillance systems, lack of proper knowledge, and unawareness have made Southeast Asia a global hotspot for antibiotic resistance^18,79^. As a Southeast Asian country, Bangladesh contributes significantly because of its poor health care standards as well as the excessive and misuse of antibiotics. Given these circumstances*,* the key findings suggest that poor knowledge, negative attitude, misuse of antibiotics, easy access to antibiotics from the pharmacy, antibiotic storage habits at home, and communication gap between parents and doctors during children's healthcare visits drive the development and spread of antibiotic resistance in the community. Based on existing scenario, it could be suggested that parents should be more aware about the use of antibiotics to their children.

However, further research is required to determine the extent of antibiotic abuse accurately. This cross-sectional study was conducted in the southern part of Dhaka City; therefore, the data collected may not be nationally representative. Finally, the interviews only covered parents attending schools in the area. Regardless of these limitations, the present study provides valuable information for assessing and improving knowledge, attitudes, and practices regarding antibiotic use among parents of school-going children.

## Conclusion

This study aimed to determine the factors that contribute to antibiotic misuse among parents. Parents’ knowledge, attitudes, and practices (KAP) on antibiotic use by their children are poor. The study identified several factors, including parents' age, sex, education level, employment status, household income, and positive attitudes, that were significantly associated with the appropriate use of antibiotics among parents. Misconceptions regarding antibiotics, their use and mechanism of action, self-medication without a doctor's prescription, purchase of antibiotics without prescriptions from pharmacies, and antibiotic storage habits at home contribute to the alarming misuse of antibiotics.

Key findings from this study will help policymakers plan and implement effective multidimensional future interventions to improve appropriate antibiotic use. This study proposes a comprehensive national initiative to address antibiotic misuse among parents of school-going children in Bangladesh. This multi-pronged approach calls for robust collaboration among policymakers, healthcare providers, and educators to address this critical public health issue. The cornerstone of the initiative lies in implementing engaging and informative parental and public education campaigns strategically disseminated across diverse platforms including television, radio, newspapers, workshops, and social media. These campaigns should aim to transcend socioeconomic barriers and effectively reach both medical professionals and the public. This study further emphasizes the crucial need for synergistic collaboration between relevant government departments to ensure a cohesive and impactful campaign. Additionally, it underscores the importance of promoting healthy lifestyle practices and leveraging the penetrative power of the mass media to disseminate vital information regarding antibiotic use. To bolster these efforts, implementation of stringent regulations governing antibiotic sales, particularly by pharmacy shop owners without prescriptions, is deemed essential. The author also highlights the paramount importance of updating and meticulously enforcing national legislation on antibiotic use. This comprehensive and multi-faceted approach aims to foster a pervasive and sustained cultural shift towards responsible antibiotic use within the community, thereby mitigating the adverse consequences of antibiotic misuse. The author also recommends conducting continuous nationwide surveillance with a community-based interventional study involving children's parents to explore the actual scenario of antibiotic use and its effects on the entire community.

## Data Availability

The datasets used and/or analyzed during the current study are available from the corresponding author upon reasonable request.
